# The First Genome Survey of the Antarctic Krill (*Euphausia superba*) Provides a Valuable Genetic Resource for Polar Biomedical Research

**DOI:** 10.3390/md18040185

**Published:** 2020-03-31

**Authors:** Yuting Huang, Chao Bian, Zhaoqun Liu, Lingling Wang, Changhu Xue, Hongliang Huang, Yunhai Yi, Xinxin You, Wei Song, Xiangzhao Mao, Linsheng Song, Qiong Shi

**Affiliations:** 1 Liaoning Key Laboratory of Marine Animal Immunology, Dalian Ocean University, Dalian 116023, China; huangyuting@genomics.cn (Y.H.); liuzhaoqun@dlou.edu.cn (Z.L.); wanglingling@dlou.edu.cn (L.W.); 2 Shenzhen Key Lab of Marine Genomics, Guangdong Provincial Key Lab of Molecular Breeding in Marine Economic Animals, BGI Academy of Marine Sciences, BGI Marine, BGI, Shenzhen 518083, China; bianchao@genomics.cn (C.B.); yiyunhai@genomics.cn (Y.Y.); youxinxin@genomics.cn (X.Y.); 3 BGI Education Center, University of Chinese Academy of Sciences, Shenzhen 518083, China; 4 Ocean University of China, Qingdao 266100, China; xuech@ouc.edu.cn (C.X.); xzhmao@ouc.edu.cn (X.M.); 5 East China Sea Fisheries Research Institute, Chinese Academy of Fishery Sciences, Shanghai 200090, China; ecshhl@163.com (H.H.); songw@ecsf.ac.cn (W.S.)

**Keywords:** Antarctic krill *(Euphausia superba)*, genome survey, mitochondrial genome, whiteleg shrimp (*Penaeus vannamei*), antimicrobial peptide (AMP), antihypertensive peptide (AHTP)

## Abstract

The world-famous Antarctic krill (*Euphausia superba*) plays a fundamental role in the Antarctic food chain. It resides in cold environments with the most abundant biomass to support the Antarctic ecology and fisheries. Here, we performed the first genome survey of the Antarctic krill, with genomic evidence for its estimated genome size of 42.1 gigabases (Gb). Such a large genome, however, is beyond our present capability to obtain a good assembly, although our sequencing data are a valuable genetic resource for subsequent polar biomedical research. We extracted 13 typical protein-coding gene sequences of the mitochondrial genome and analyzed simple sequence repeats (SSRs), which are useful for species identification and origin determination. Meanwhile, we conducted a high-throughput comparative identification of putative antimicrobial peptides (AMPs) and antihypertensive peptides (AHTPs) from whole-body transcriptomes of the Antarctic krill and its well-known counterpart, the whiteleg shrimp (*Penaeus vannamei*; resident in warm waters). Related data revealed that AMPs/AMP precursors and AHTPs were generally conserved, with interesting variations between the two crustacean species. In summary, as the first report of estimated genome size of the Antarctic krill, our present genome survey data provide a foundation for further biological research into this polar species. Our preliminary investigations on bioactive peptides will bring a new perspective for the in-depth development of novel marine drugs.

## 1. Introduction

The Antarctic krill (*Euphausia superba*), widely distributed in the Southern Ocean, provides the most abundant biomass for Antarctic ecology and fisheries [1]. It establishes a critical link between primary producers (phytoplankton) and apex predators (such as fishes, squids, penguins, and seals) in the Antarctic food chains [2,3], with an estimated biomass of 100~500 million tons [3]. With such a large number of Antarctic krill, the Southern Ocean supports an unprecedented abundance of upper trophic-level predators. Field observations have reported that population trends of some krill predators are in part influenced by the abundance changes in Antarctic krill [4]. Human beings are also benefited by many extracted products from the Antarctic krill, such as pharmaceuticals, nutraceutical health foods, and aquaculture feeds [5]. Thus, getting insight into the genetic resources of the Antarctic krill is necessary for species protection, as well as for the development of related fisheries and industry. Studies on the genetic resources of the Antarctic krill primarily focus on transcriptomes [1,3], simple sequence repeats (SSRs) [1], and the mitochondrial genome [6,7]. These data provide valuable foundation for in-depth genetic research on this polar species. However, no complete genome assembly is available for this important crustacean.

Recently, a high-quality genome assembly of its famous counterpart, the whiteleg shrimp (*Penaeus vannamei*), was published [8]. As we know, this shrimp species predominantly inhabits tropical and subtropical areas, and has been extensively cultivated in Asian countries. Resident in such remarkably different environments, the Antarctic krill and the whiteleg shrimp may have undergone differential genetic variances. Since the genome size of the Antarctic krill is huge (over 40 Gb; see more details in our Results), we had to stop our genome project temporarily with only a genome survey available. However, these genomic data are still useful for uncovering short sequences, such as bioactive peptides encoded by entire or partial genes.

For example, antimicrobial peptides (AMPs) are short with broad-spectrum antimicrobial activities; most of them can be classified into either own gene type or proteolysis type (derived from immune related genes) [9]. They are usually less than 10 kDa while acting as the major components of the innate immune defense system in marine invertebrates [10,11], and habitat-related variances in AMPs/AMP precursors are likely to exist in various crustaceans [11]. Although AMPs in the Antarctic krill have received some attention [12], researchers still know little of the overall AMPs in such an important crustacean species and other marine animals from diverse habitats. Looking into the connection between AMPs/AMP precursors and creatures from various environments may help us to identify novel peptides and even apply them for species protection and human health.

Angiotensin converting enzyme (ACE) inhibitors are preferred antihypertensive drugs, and antihypertensive peptides (AHTPs), another important representative with short sequences, are the most effective and popularly studied ACE inhibitory peptides [13]. A lot of AHTPs are usually digested from natural products, and they most frequently contain 2~10 amino acids. Endogenous AHTPs can be hydrolyzed and degraded with assistance of digestive enzymes from in vivo proteins; by binding to ACEs or related receptors, they may adjust the renin angiotensin system for antihypertensive effects [14]. In recent years, several AHTPs have been isolated from Antarctic krill, and some of them have been used for mechanism studies [15,16]. However, general knowledge of *E. superba* AHTPs from the genomic or transcriptomic perspective is still limited. Comparative investigations on AHTPs between the Antarctic krill and the whiteleg shrimp may make contribution to our better understanding of AHTPs in various animals and identification of candidates for practical utilization as pharmaceuticals.

In the present study, we performed the first genome survey of the Antarctic krill. Such a large genome, however, is difficult for us to obtain a good assembly, although our sequencing data are a valuable genetic resource for subsequent polar biomedical research. Partial mitochondrial genome and many SSRs were able to be extracted for species identification and origin determination. Moreover, a comparative study on AMPs and AHTPs, based on available transcriptome and genome sequences from both the Antarctic krill and the whiteleg shrimp, was conducted. Here, our main aim is to establish a basic genomic and genetic foundation for future polar biomedical studies on the Antarctic krill, especially to initiate a preliminary exploration of bioactive peptides in a polar animal for the development of novel marine drugs.

## 2. Results

### 2.1. A Genome Survey of the Antarctic Krill

**Figure 1:**
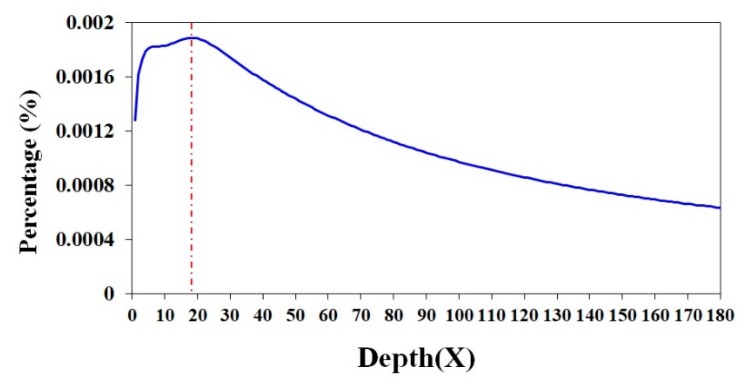
A 17-mer distribution curve of the Antarctic krill (*E. superba*). The *x*-axis is the sequencing depth (X) of each unique 17-mer, and the *y*-axis is the percentage of these unique 17–mers.

In total, we obtained 911.0 Gb of raw reads sequenced by a BGISeq500 platform (BGI-Shenzhen, Shenzhen, China) from all the constructed libraries (400 bp in length). A detailed K-mer analysis [14] was performed to estimate the genome size, and a survey peak was visible with high heterozygosity in Antarctic krill (see Figure 1). We calculated the genome size (G) of the Antarctic krill according to the following formula: G = K_num/K_depth [17]. In our present study, the total number of K-mers (K_num) was 758,531,899,196 and the K_depth was 18 (Table 1 and Figure 1). Therefore, we estimated that the genome size of *E. superba* was 42.1 Gb; the sequencing depth (X) of the clean data is therefore ~21 of the estimated genome size (Table 1).

### 2.2. Assembly of Extracted Partial Mitochondrial Genome

A roughly complete mitochondrial genome of the Antarctic krill was assembled to be 12,272 bp in length, while the entire length was 15,498 bp in a previous report [6]. Based on these sequences, we extracted all the 13 typical mitochondrial protein-coding genes from our genomic raw sequences, although certain gene sequences are still partial (see File S1). These genes include cytochrome c oxidase subunit I (*coxI*), cytochrome c oxidase subunit II (*coxII*), cytochrome c oxidase subunit III (*coxIII*), ATPase subunit 8 (*atp8*), ATPase subunit 6 (*atp6*), NADH dehydrogenase subunit 3 (*nad3*), NADH dehydrogenase subunit 5 (*nad5*), NADH dehydrogenase subunit 4 (*nad4*), NADH dehydrogenase subunit 4L (*nad4L*), NADH dehydrogenase subunit 6 (*nad6*), cytochrome b (*Cytb*), NADH dehydrogenase subunit 1 (*nad1*), and NADH dehydrogenase subunit 2 (*nad2*). More details about the gene map can be seen in Figure S1, in which the order of these genes was arranged manually, in accordance with the previous report [6].

#### 2.2.1. Annotation and Analysis of Our Extracted Mitochondrial Genes

These mitochondrial genes were assigned into six Kyoto Encyclopedia of Genes and Genomes (KEGG) pathways (see Tables S1 and S2) by using the BLASTp [18] to map against the public KEGG database [19]. Energy metabolism pathway, as the representative one, consists of 11 genes (Table S2), such as *nad5*, *nad4*, *nad1*, *coxI*, and *coxII* (Figure S2A). The functions of these extracted mitochondrial genes were predicted with classifications by searching the public Gene Ontology (GO) databases [20]. Based on the GO annotation, we assigned them into 13 subcategories under three main categories, including biological process (3), cellular component (7), and molecular function (3). The “catalytic activity” terms (8; 53.3%) were obviously dominant in the “molecular function” (Figure S2B). 

#### 2.2.2. Multiple Sequence Alignment and Phylogenetic Analysis of the Representative Mitochondrial Gene *nad4L*

**Figure 2:**
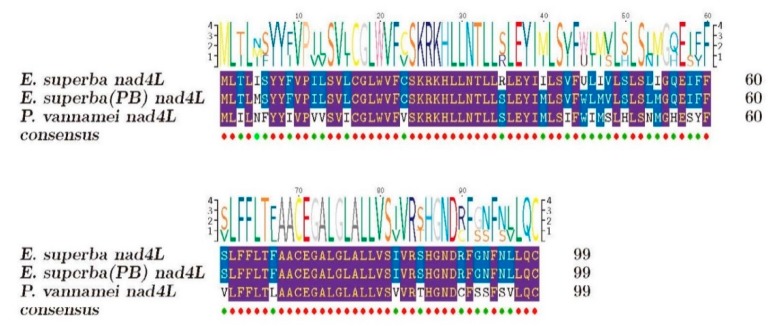
Multiple sequence alignment of the putative *nad4L* genes. Red circles at the bottom stand for the same residues. Blue and purple colors on the sequences represent the alignment with identity >50% and >80%, respectively.

The representative mitochondrial gene *nad4L* from both Antarctic krill and whiteleg shrimp (a good counterpart from warm waters) were chosen to perform multiple sequence alignment (Figure 2). We observed 6 and 30 different residues between the Antarctic krill in this study and the sample collected from Prydz Bay (*E. superba* PB) [6], and between our Antarctic krill and the whiteleg shrimp, respectively. Obviously, both Antarctic krill samples were more conserved; however, their sequence variances may represent various origins. 

**Figure 3:**
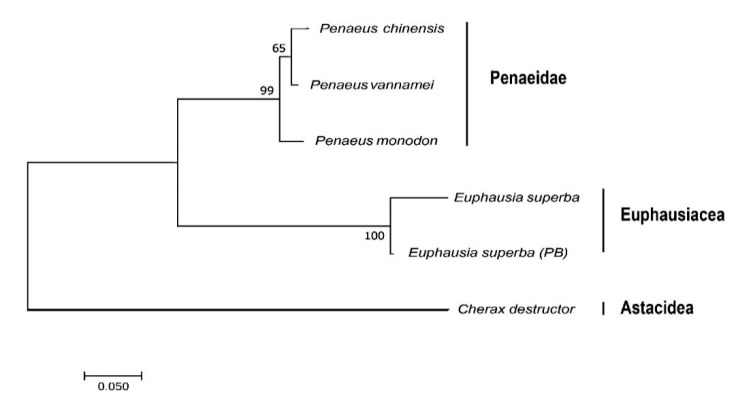
Phylogenetic topology of *nad4L* derived from the Neighbor–Joining method [21]. The bootstrap test employed 1,000 replicates, and the numbers next to branches were replicate percentage of taxa clustering [22]. Corresponding amino acid sequences were analyzed in MEGA7 [23].

To confirm the Antarctic krill in the present study is the same species as reported *E. superba* (PB) [6] and to provide more evidence for the phylogenetic relationship between Penaeidae and Euphausiacea, we used the *nad4L* sequence of Australian freshwater crayfish (*Cherax destructor*; NCBI Gene ID: 2827710) as an out-group and constructed a phylogenetic tree of *nad4L* among the Antarctic krill, the whiteleg shrimp, and several other representative shrimps. The established phylogenetic topology was divided into two main groups of Penaeidae and Euphausiacea (Figure 3). The *nad4L* identified for the Antarctic krill in the present study was not surprised to be much closer to the reported *E. superba* (PB)’s [6]. That is to say, the *nad4L* is practicable for the Antarctic krill in species identification and has potential for origin determination.

### 2.3. Assemblies of Reported Transcriptomes of the Antarctic Krill and the Whiteleg Shrimp

Raw data of the Antarctic krill transcriptomes were downloaded from the National Center for Biotechnology Information (NCBI; accession number PRJNA307639). Total RNA was isolated from six whole specimens that were collected from the Southern Ocean. High-throughput transcriptome sequencing (pair-ended at 2 × 150 bp) on an Illumina HiSeq 3000 platform generated ~77.9 million of raw reads, equal to 11.8 Gb [1]. Here, we assembled these available public transcriptome sequences. After removal of low-quality reads and trimming adapter sequences, we collected 10.6 million of clean reads corresponding to 1.5 Gb, and generated 16,797 unigenes with a GC rate of 37.6% for the Antarctic krill. As summarized in Table 2 for the transcriptome assembly, the average length was 637 bp and the N50 was 923 bp. 

The raw data of the whiteleg shrimp transcriptomes were downloaded from NCBI under the accession number PRJNA288849. In the corresponding report [24], whole-body adult shrimps at three molting stages (including inter-molt, pre-molt and post-molt) were collected from a laboratory culture, and an Illumina HiSeq 2500 platform was used for the sequencing of cDNA libraries. Here, we assembled the publicly available transcriptome sequences. Finally, a total of 90.9 million clean reads (equal to 3.1 Gb) were obtained after data filtering. In the transcriptome assembly, 3,768 unigenes were annotated; the average length was 574 bp and the N50 value was 759 bp, with an average GC content of 51.0% (Table 3).

These assemblies of reported transcriptomes were set for high-throughput SSR identification in the Antarctic krill (Section 2.4) and further comparisons of AMPs and AHTPs between the Antarctic krill and the whiteleg shrimp (Section 2.5 and Section 2.6).

### 2.4. High-throughput SSR Identification in the Antarctic Krill

In order to investigate whether genomic data of the Antarctic krill can be used for the development of genetic markers for species identification and origin determination, we picked SSR as a trial example. Interestingly, a total of 1,026 and 74,661 SSRs were identified from our transcriptome (Section 2.3) and partial genome raw data (Section 2.1) of the Antarctic krill, respectively (Table S3). These SSRs ranged from 2 to 6 bp. 

**Figure 4:**
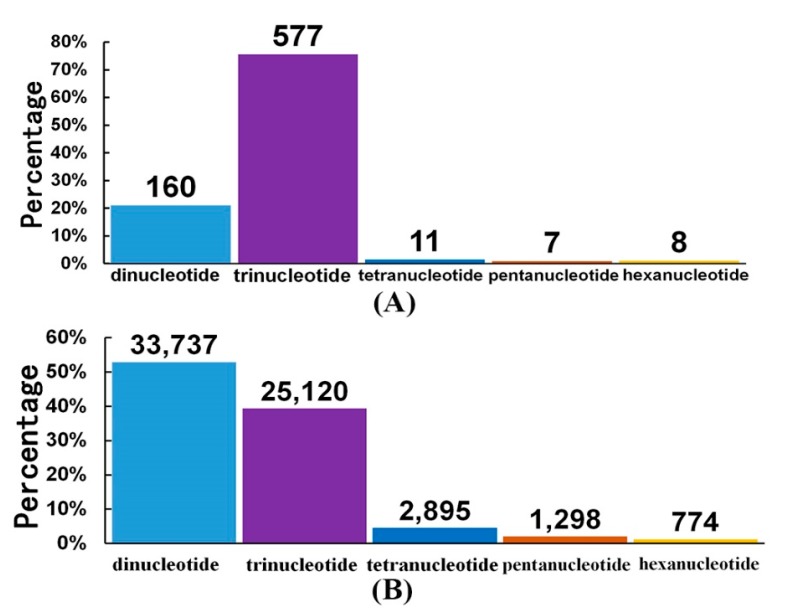
SSR classification in the Antarctic krill. Data were analyzed in our transcriptome assembly (**A**; Section 2.3) and our partial genome raw data (**B**; Section 2.1). The *x*-axis is the nucleotide type of each SSR, and the *y*-axis is the percentages of these SSRs. The number on the top of each bar is the total amount of corresponding SSRs.

In the transcriptome assembly, the most abundant type of SSRs was the trinucleotide repeats. As shown in Figure 4A, the total number of SSRs with trinucleotide repeats was 577, and their percentage reached 75.62%; the second highest number of SSRs, 160, was with dinucleotide repeats. However, in our partial genome raw data, the situation seemed to be different. The most abundant SSRs were with dinucleotide repeats (33,737), accounting for 52.9%; SSRs with trinucleotide repeats were dropped down to the second highest number, 25,120 (see more details in Figure 4B).

### 2.5. Comparisons of AMPs between the Antarctic Krill and the Whiteleg Shrimp

**Figure 5:**
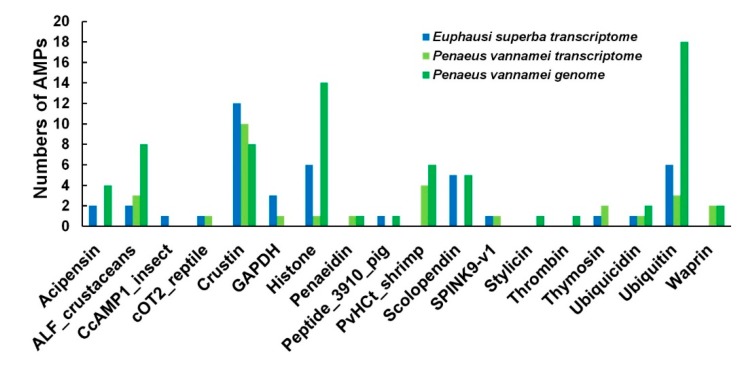
Summary of the identified anti-microbial peptides (AMPs)/AMP precursors from the Antarctic krill transcriptome and the whiteleg shrimp transcriptome and genome assemblies. Blue bars represent those identified in the former (*E. superba*), and green bars represent those retrieved from the latter (*P. vannamei*).

Employing our previously collected list of active AMPs (Table S4) and analysis pipeline [9], we employed BLAST to search the Antarctic krill and the whiteleg shrimp transcripts (Section 2.3) and identified 85 and 78 putative AMPs (Table S5), respectively. These AMPs/AMP precursors were classified into 16 groups (Figure 5). Interestingly, in the present study, CcAMP1_insect was only identified in the Antarctic krill transcripts, but not in the transcriptome and genome sequences of the whiteleg shrimp (the third group in Figure 5). We also noted that histone 2 (one of the six histones; with the mapped AMP of Buforin I) and ubiquitin/ribosomal S27 fusion protein (with the mapped AMP of cgUbiquitin) were the top two AMP precursors with the highest transcription values in the Antarctic krill (Table S6; not detectable in the whiteleg shrimp). PvHCt, corresponding to the C-terminal fragment in hemocyanin of *P. vannamei* [25], presented high transcription values in our assembled whiteleg shrimp whole-body transcriptomes (see Table S6).

**Figure 6:**
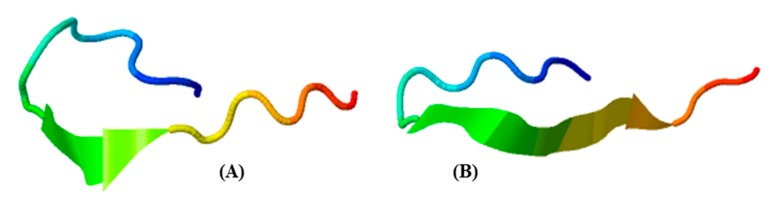
Predicted 3D structures of CcAMP1_insect in insect *C. chinensis* (**A**) and the Antarctic krill (**B**). They were predicted by I–TASSER with high confidence (see more details in Section 3.2).

Meanwhile, we observed that the homologous sequence of the CcAMP1_insect extracted from the Antarctic krill transcriptomes in the present study had one different residue (K) from that of insect *Coridius chinensis*’s (V) (Figure 6). The predicted 3D structure of *E. superba* CcAMP1_insect (Figure 6B) was different from the *C. chinensis*’s (Figure 6A), although both contained strands and coils.

**Figure 7:**
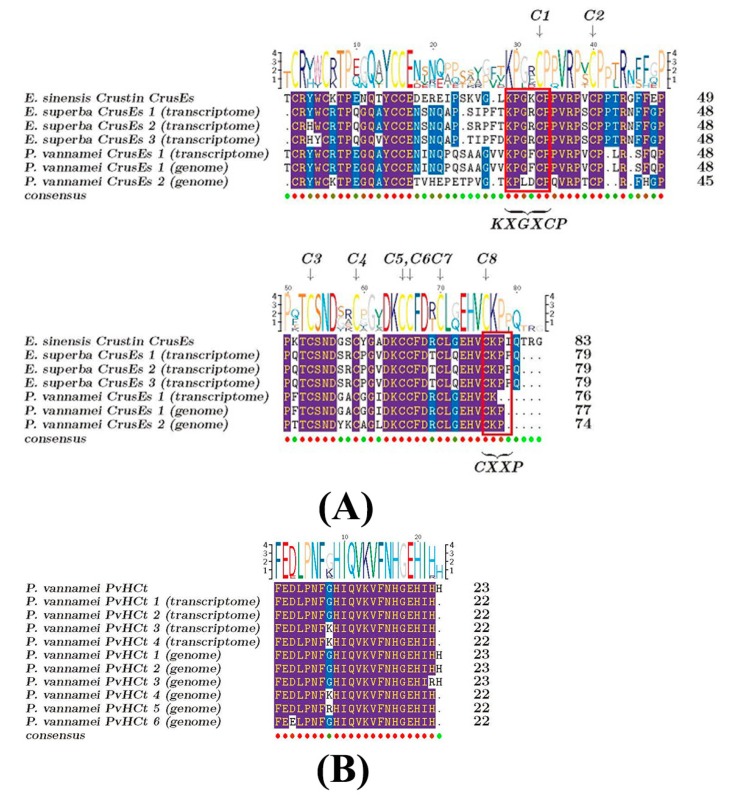
Multiple sequence alignment of representative AMPs/AMP precursors. (**A**) Crustins from different species. The eight cysteine residues, conserved in all crustaceans with the consensus sequences of whey-acidic proteins [26], were also present in the CrusE sequences, as indicated by arrows and C1~C8. (**B**) PvHCt from the whiteleg shrimp (*P. vannamei*). Red circles at the bottom stand for the same residues. Blue marks represent the alignment with identity >50%.

An important AMP category, crustin, abundantly existed in both crustaceans (Figure 5). Some of them, named CrusEs, belong to a group of cysteine-rich antibacterial peptides with whey acidic protein (WAP) domains, including two four-disulfide core domains and each domain with 8 conserved cysteine residues. The WAP domains also contain a KXGXCP motif [26,27]. Multiple sequence alignments of crustin (CrusEs; Figure 7A) demonstrated that both the Antarctic krill and the whiteleg shrimp possessed conserved cysteines. Another CXXP motif of the WAP domain could also be identified in the Antarctic krill, while it was incomplete in the whiteleg shrimp (see the detailed *P. vannamei* CrusEs (genome) sequence in Figure 7A). A phylogenetic analysis of the representative CrusEs between the two crustaceans was performed for more comparison (Figure S3; data from Figure 7A).

The transcription levels of crustin (such as CrusEs and MrCrs) were usually very high in the Antarctic krill, whereas they were not detectable in the whiteleg shrimp (see more details in Table S6). Interestingly, we observed three CrusEs in the former but only one in the latter. Similarly, another crustin category (MrCrs) was highly transcribed in the Antarctic krill but not detectable in the whiteleg shrimp; there were nine MrCrs in the former but only two in the latter (Table S6). There were also some *P. vannamei* unique crustins including Crustin*Pm*1, Crustin*Pm*7 as well as other AMPs including CqCrs, SWD*Pm*2, PvHCt, penaeidin, and waprin were obtained from the whiteleg shrimp transcriptome and genome, although their transcriptions were not detectable either (see Table S6). We noted that sylicin and thrombin were not identified in the transcriptome but only in the genome of the whiteleg shrimp in this study (Figure 5, Table S6). As expected, penaeidins of the whiteleg shrimp were the same as those of the reported penaeid shrimp [28]. The sequence of PvHCt identified in the present study was also highly conserved in comparison to that in previous studies [25] with minor variances (Figure 7B). 

### 2.6. Prediction and Analysis of AHTPs in both Crustacean Species

**Figure 8:**
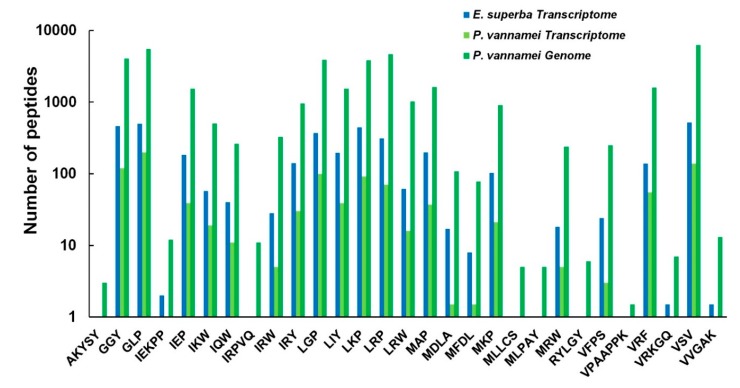
A comparative overview of the identified antihypertensive peptides (AHTPs) in both crustaceans. Blue bars denote the AHTPs identified from *E. superba* transcriptome [1]; green bars represent the *P. vannamei* AMPs retrieved from both transcriptome [24] and genome [8] data.

To identify potential AHTPs in the translated proteomes (from the genome or transcriptome data) of the Antarctic krill and the whiteleg shrimp, we built a local AHTP-searching database [13]. Most of the known AHTPs searched in this database have been verified in reported studies, and they are usually tripeptides with less than 10 amino acids; the top 50 AHTPs with the highest activity were chosen to identify AHTPs in the two crustacean species, as reported in our previous study [14]. Finally, in the Antarctic krill, 23 AHTP sequences were identified from the transcriptomes; in the whiteleg shrimp, AHTP numbers were 20 and 29 from the reported transcriptomes [1] and genome assembly [8] (Table S9). The detailed location(s) of each matched AHTP sequence in its corresponding protein was listed in Table S7. It seems that AHTPs are almost overlapped between the two crustaceans (Figure 8, Table S9). 

In the Antarctic krill, we observed that involucrin had the most abundant AHTP hit numbers (14; Table S8). In the whiteleg shrimp, however, it is the collagen alpha-1 chain-like protein that possessed the most AHTP hits (27; see Table S8). As shown in Figure 8, the richest AHTP categories were VSV, GLP, LRP, GGY, LGP, LKP in both crustacean species. Some AHTPs were identified only in the genome of whiteleg shrimp but were not detectable in the transcripts of Antarctic krill and whiteleg shrimp, such as AKYSY, IRPVQ, MLLCS, MLPAY, RYLGY, as well as VPAAPPK (Figure 8, Table S9).

## 3. Discussion

### 3.1. Importance to Characterize the Genome Size, Mitochondrial Genome and SSRs in the Antarctic Krill

Our genome survey in the present study first calculated the estimated genome size of the Antarctic Krill as 42.1 Gb (Figure 1). This result is consistent with a previous report of 47.5 Gb from both flow cytometry and Feulgen image analysis densitometry [29]. However, such a large genome is beyond our present capacity to obtain a good assembly. We had to stop our genome project temporally, without wasting more time and money, until we figure out practicable assemble strategies.

Mitochondrial genes have been widely used for species identification. In the present study, sequence alignment and phylogenetic analysis of the representative *nad4L* between two sources of Antarctic krill and the whiteleg shrimp (Figure 2 and Figure 3) revealed the phylogenetic relationship between Penaeidae and Euphausiacea (Figure 3). We also mapped the 13 typical protein-coding genes (Figure S1), which were similar to a previous report in *E. superba* (PB) [1]. It seems that the extraction of mitochondrial genes from the genome survey data can be used for origin determination, which will be informative for in-depth studies on various sources of Antarctic krill. Meanwhile, the mitochondrial roles for cold adaptation have been studied in cod. The overall COX activities in liver were reported to be higher in the cold-adapted population, although they were not affected by cold acclimation [30]. Similarly, the mitochondrial genes in the Antarctic krill may also benefit from the examination of cold adaptive mechanisms to polar environments. 

SSRs, also called microsatellite markers, are a class of repetitive DNA sequences. They usually consist of tandem repeating units of mon-, di-, tri-, and tetra-nucleotide types. They have become well-known markers for identifying and classifying species from various resources [31]. In diverse shrimps, SSRs have been developed for potential applications in genetic studies, kinship analysis, origin determination, etc. [32]. Dinucleotide repeats were the most abundant type in reported Antarctic krill transcriptomes [1] and our partial genome raw data (Figure 4B). With the availability of more genome-wide SSRs from other resources of Antarctic krill, we may determine the origin of any commercial frozen products in the future.

### 3.2. Similarities and Differences of AMPs between the Two Examined Crustacean Species

Our present study also provided a valuable genetic resource for AMP comparisons between Antarctic krill and whiteleg shrimp. It seems that AMPs and AMP precursors were generally conserved, while being differentially various between the two crustacean species, possibly due to their residency in significantly different waters.

The Antarctic krill unique CcAMP1 was originally extracted from *C. chinensis* [33]. However, there is no document revealing the correlation of CcAMP1 and environmental adaptation. According to previous reports, there were five hydrophobic amino acids (VAWVL) on its surface, which might construct an α-helix to destroy the cell member integrity of bacteria [25] for stronger antimicrobial effects. However, since the predicted 3D structures of CcAMP1 of *E. superba* and *C. chinensis* in the present study were different (without the critical helix structures; Figure 6), thereby leading to uncertainty of the putative antimicrobial activity. As highly transcribed in the Antarctic krill, a specific protease was reported to be responsible for the generation of AMP buforin I from the histone 2 [34]. Buforin I was originally identified from Asian toad and showed strong antimicrobial activities against a broad spectrum of bacteria [35]. The AMP cgUbiquitin, mapped in the ubiquitin/ribosomal S27 fusion protein, was originally isolated from the gill of the Pacific oyster, and its precursor mRNA was reported to be significantly upregulated after *Vibrio* stimulation [36]. We therefore propose that these Antarctic krill’s unique and highly transcribed AMPs were possibly important for the survival of aquatic animals in a cold environment.

For the crustin (CrusEs) from both Antarctic krill and whiteleg shrimp, the CXXP motif was identified completely from the former, while incomplete in the latter. This may cause non-functionality or neo-functionality in the whiteleg shrimp. Furthermore, the cDNA sequences of CrusEs, previously identified from Chinese mitten crab with the validation of purified proteins to inhibit the growth of Gram-positive bacteria [26], and the cDNA encoding MrCrs, with a first report in a freshwater prawn *Macrobrachium rosenbergii*, could be inductively expressed when the host was affected by bacteria [37]. Their high transcription levels and the greater number present in Antarctic krill compared to whiteleg shrimp suggest their more important roles in Antarctic krill.

The whiteleg shrimp’s unique AMPs, identified in the present study, were from a preliminary exploration. It will be necessary to perform a double check when the genome assembly of the Antarctic krill is available. Crustin*Pm*1, crustin*Pm*7, CqCrs, and stylicin were previously identified from black tiger shrimp (*Penaeus monodon*), red claw crayfish (*Cherax quadricarinatus*) and Pacific blue shrimp (*Litopenaeus stylirostris*), and exhibited antimicrobial activities against bacterial and fungal invasions [37,38,39]. SWD*Pm*2, originally identified from hemocytes of the black tiger shrimp, is another group of WAP domains containing protein similar to crustin; it was up-regulated after an injection of white spot syndrome virus (WSSV) [40]. PvHCt, a histidine-rich antimicrobial peptide with antimicrobial activity to fungal cells, was originally found in whiteleg shrimp. It has potentially been derived in large quantities by the proteolytic cleavage of the hemocyanin protein [25]. Unlike gene-encoded cationic defense peptides such as crustins and penaeidins, PvHCt can be obtained massively by hemocyanin proteolytic cleavage without any recombinant production or purification system and is the most abundant plasma protein in crustaceans [25]. The whiteleg shrimp’s unique AMPs identified in our present study were potentially more important for this species. Furthermore, those genes with high transcription levels, such as AMP precursor histone 2, ubiquitin/ribosomal S27 fusion protein, and the WAP-domain-containing proteins including crustin and waprin, as well as hemocyanins-derived PvHCt, may be promising antimicrobial candidates and potentially good sources for the development of AMP-based drugs. Regarding the stylicin [39] and thrombin [41], which were only identified in the *P. vannamei* genome in our present study, we guess that they may not be well transcribed.

Meanwhile, the environmental specificity of the AMPs in both Antarctic krill and whiteleg shrimp were also investigated. Interestingly, we found that some AMPs that are unique to the Antarctic krill or in both crustaceans may not show environmental specificity, because the habitats of their derived species and the bacteria inhibited by them were not coincident with Antarctic krill’s distribution areas [35,36,38,42,43]. However, some AMPs only identified in whiteleg shrimp in the present study (such as crustin*Pm*1) may play important roles in special responses to warm environments due to the fact that their originally sourced species were geographically consistent with whiteleg shrimp [44].

### 3.3. Conservations of AHTPs between the Two Crustacean Species

AHTPs were investigated in the present study for the potential development of antihypertensive drugs from the Antarctic krill. Although from different environments, unlike AMPs, the most abundant AHTPs (such as VSV) were the same, and most of the AHTPs can be found both in the datasets of the Antarctic krill as well as the whiteleg shrimp. These data indicate that the AHTPs may be highly conserved in both crustaceans, which is consistent with our previous report of marine mammals [14]. However, those AHTPs identified only in the genome of the whiteleg shrimp also need to be retrieved from the genome of the Antarctic krill once its good assembly is made available in future.

Interestingly, the AHTP mapping ratio in involucrin was higher than other proteins in the Antarctic krill, with AHTP hits to 14. The Involucrin with the most abundant AHTPs is a keratinocyte protein [45], and keratinocytes is the major constituent of the epidermis tissue [46] of the skin’s outer layer [47]. As for the whiteleg shrimp, however, both collagen alpha-1(V) chain-like protein and collagen alpha-1(XI) chain-like protein were the most abundant AHTP-containing proteins, which were revealed previously by us in fishes [13]. In fact, ACE-inhibitory peptides had been obtained from the collagen hydrolysates of many animals [48,49,50,51,52,53]. Based on our present work, we propose to apply the epidermis tissues of the Antarctic krill and collagen alpha chain proteins of shrimps as a promising resource to obtain AHTP production. Of course, similarly to the AMPs, the ACE inhibitory activity can be evaluated after the comprehensive prediction results are available, once the genome of the Antarctic krill is assembled with high quality.

## 4. Materials and Methods

### 4.1. Genomic DNA Extraction and Genome Sequencing for the Antarctic Krill

Our experimental procedures complied with the current laws on animal welfare and research in China. Alive Antarctic krill specimens were collected from the Argentine Sea area (45°92’S,61°82’W). Genomic DNAs were extracted from the whole bodies of pooled specimens with a Qiagen GenomicTip100 kit (Qiagen, Germanton, MD, USA) according to the manufacturer’s protocol. With the traditional whole-genome shotgun sequencing strategy [14], we used 1 μg of normalized DNA to prepare a paired-end short-insert library (400 bp). Quantification and size estimations of the library were performed on a Zebra Flowcell 3.1 chip. Finally, the library was normalized to 15 ng/μL for paired-end sequencing (100 bp in length) on a BGISeq500 platform (BGI, Shenzhen, China). Raw genome sequencing reads have been deposited in the NCBI and China National GeneBank (CNGB) under the project IDs PRJNA598052 and CNP0000808, respectively.

### 4.2. Assembly of the Antarctic Krill Mitochondrial Genome and Transcriptomes

At first, we estimated the genome size of the Antarctic krill using a routine K–mer analysis method [14] with the following formula: G = K_num/K_depth, where K_num is the total number of K–mers, and K_depth indicates the frequency of reads occurring more frequently than others [17].

BGI paired-end reads were filtered with SOAPnuke1.5.6 [54] and the common adapter sequences were trimmed. A roughly complete mitochondrial genome of the Antarctic krill was assembled. Firstly, the mitochondrial genome of a congeneric *E. superba* (downloaded from the NCBI with an accession number EU583500.1) was employed [6]. Those sequencing reads with a high similarity to the reference mitochondrial genome were identified by SOAP2 (version 2.21) [55]. Subsequently, SPAdes (version 3.10.0) was employed [56] to assemble all these highly similar reads. Finally, Blast (version 2.6.1) [57] was applied to compare the archived assembly with the reference mitochondrial genome. The scaffolds containing a low length (<200 bp) were removed and the filtered scaffolds were combined into one sequence as a mitochondrial genome sequence. The redundancy sequences were also manually deleted. Then, the mitochondrial genome sequence was annotated with AGORA [58] to get the 13 protein-coding genes nucleotide sequences.

The *E. superba* and *P. vannamei* transcriptome sequences were downloaded from the NCBI with accession numbers PRJNA307639 (SRR3089571) [1] and PRJNA288849 [24], respectively. SOAPnuke 1.5.6 [54] was employed to filter the paired-end short reads of transcriptomes with the removal of contaminants, adapters and those low-quality reads (with over 5% non-sequenced bases or more than 20% of bases with quality score ≤ 10). We then employed Trinity v2.5.1 [59] to assemble the remaining clean reads, which were clustered by using TGICL v2.1 [60] based on sequence similarity and assembled to consensus unigenes. Clean reads were aligned to the de novo assemblies with a Bowtie 2 read aligner [61] to calculate gene transcription values in the assembled transcriptomes. Finally, we used RNA–Seq by Expectation Maximization (RSEM) v1.2.31 [62] to estimate transcript abundance in term of FPKM (fragments per kilobase of transcript per million mapped reads) values. The candidate coding regions from the assembled transcripts were identified with TransDecoder (http://transdecoder.sourceforge.net/), and then were translated into amino acid sequences using the standard codon table.

### 4.3. Functional Annotation of the Extracted Mitochondrial Genome and the Reported Transcriptomes

The amino acid sequences of Antarctic krill mitochondrial protein-coding genes from AGORA [58] annotation results were mapped to KEGG [19] pathway annotations using Diamond [18] with an *E*-value threshold of 1.0 × 10^−5^. Blast2GO v4.1 [63] was employed to perform GO [20] annotation of NCBI Nr blast results. Unigene sequences from the Antarctic krill and the whiteleg shrimp transcriptomes were searched using Diamond [18] and blastn [57] against the NCBI Nr and UniProtKB/Swiss-Prot [64] databases (*E*–value ≤ 1.0 × 10^–5^) to retrieve protein functional annotations based on sequence similarity.

### 4.4. Phylogenetic Analysis and Multiple Sequence Alignment

Along with the *nad4L* sequence extracted from the Antarctic krill mitochondrial genome, we also extracted several other malacostracan *nad4L* sequences from the NCBI for a subsequent phylogenetic analysis. Examined species include *Penaeus chinensis, P. vannamei, P. monodon*, *E. superba* (from the present study)*, E. superba* (PB), and *Cherax destructor* (as the out group). The translated protein sequences from these species were aligned using mafft v7.158b [65] with default parameters. The phylogenetic tree was constructed with the Neighbor Joining (NJ) of pairwise distances using MEGA 7.0 [23]. Multiple sequence alignment was performed using MEGA 7.0 [23], and the archived results were further analyzed and visualized by TEXshade (version 2.12.14) [66].

### 4.5. SSR Analysis

We employed our own script SSR.sh to search known SSRs from the reported transcriptome and our randomly selected partial genome raw data of the Antarctic krill. Our own script filter_ssr.pl was used to calculate SSR ratio, and the SSR distribution map was plotted with our own script draw_ssr.pl and Excel. 

### 4.6. AMP Analysis 

A total of 3073 AMP sequences were used as a local AMPs searching list as previously reported [9,67]. Standard homology searches were performed against the *E. superba* [1] and *P. vannamei* transcriptomes [24] as well as *P. vannamei* genome [8] to predict putative AMP sequences. In brief, index transcriptome and genome databases were built by running a makeblastdb command in ncbi-blast-2.6.0 [57]. Subsequently, the tBLASTn (*E*-value of 1.0 × 10^–5^) in ncbi-blast-2.6.0 [57] was employed to search our reference AMP list from the index transcriptome and genome databases with filtering of those with a query align ratio less than 0.5. 

### 4.7. AHTP Analysis

The AHTPs with the top 50 inhibitory activities were compiled as a local searching reference as described in our previous study [13]. Employing a local custom Perl script pipeline, we identified matched AHTPs sequences and locations from target proteins in the transcriptomes of *E. superba* [1] and *P. vannamei* [24], as well as the genome of *P. vannamei* [8]. AHTPs hit numbers in the mapped proteins were summarized for comparison between the two crustacean species. 

### 4.8. Tertiary Structure Prediction

To predict the 3D structures of AMPs, I–TASSER [68] was employed and the high confidence model is supported by high C-score. The top ten starting threading templates for the predicted 3D structures of CcAMP1_insect in *C. chinensis* were 3hiaB, 5lqwX, 1jy4A, 3mlqE, 6et5A, 1jy4A, 3hiaB, 1e0nA, 1jy4A and 3hiaB, and the first starting template was 3HIA, a crystal structure of the choline-binding domain of Spr1274 in *Streptococcus pneumoniae*. The top ten starting threading templates for the CcAMP1_insect in the Antarctic krill were 5af7A, 5lqwX, 1jy4A, 3mlqE, 1udyA, 1jy4A, 3hiaB, 6pz9D, 5jscA and 3hiaB, and the first starting template was 5AF7 for the 3-Sulfinopropionyl-coenzyme A (3SP-CoA) desulfinase from *Advenella mimigardefordensis* DPN7T: crystal structure and function of a desulfinase with an acyl-CoA dehydrogenase fold. In our present work, the C-scores with a range between –5 and 2 were collected as confidence indexes for model estimation. 

## 5. Conclusions

At first, we reported a genome survey of Antarctic krill, the most fundamental animal in the Antarctic food chain. Partial mitochondrial genome and abundant SSRs were extracted from our archived partial genome raw data and reported transcriptomes, which may be useful for the species identification and origin determination of this important polar crustacean species. A high-throughput identification and comparison of AMPs/AMP precursors and AHTPs between Antarctic krill and its famous counterpart, the whiteleg shrimp from warm waters, revealed general conservation with interesting variations between the two species. In summary, as the first report of the estimated genome size of Antarctic krill, our present genome survey data provide a foundation for further biological research of this economically and ecologically important invertebrate species. Our primary investigations on bioactive peptides (including AMPs and AHTPs) on a large-scale from such a polar species will bring new a perspective for in-depth predictions and the development of novel marine drugs in the future.

## Figures and Tables

**Table 1:** Statistics of 17–mers for the genome size estimation.

K-mer	K_num	K_depth	Genome Size	Clean Base (bp)	Depth (X)
17	758,531,899,196	18	42,140,661,066	902,660,212,000	21

**Table 2:** Summary of our de novo assembly of the previously reported *E. superba* transcriptomes [1].

Parameter	Value
Total Number (unigene)	16,797
Total Length (bp)	10,715,598
Mean Length (bp)	637
N50 (bp)	923
GC (%)	37.63

**Table 3:** Summary of our de novo assembly of the reported *P. vannamei* transcriptomes [24].

Parameter	Value
Total Number (unigene)	3,768
Total Length (bp)	2,165,058
Mean Length (bp)	574
N50 (bp)	759
GC (%)	50.95

## Supplementary Materials

The following are available online at https://www.mdpi.com/1660-3397/18/4/185/s1. Figure S1. A sketch map for the 13 mitochondrial protein-coding genes of the Antarctic krill. The orders of these genes are in a clockwise direction, except for *nad1*, *nad4*, *nad4L*, and *nad5*. Figure S2. Functional classification of the *E. superba* mitochondrial genome. Figure S3. A phylogenetic analysis of the representative AMP precursors, CrusEs, from both crustaceans. File S1. Representative sequences of the 13 mitochondrial protein-coding genes of the Antarctic krill, using the reported entire mitochondrial genome sequence as the reference. Table S1. KEGG analysis of the Antarctic krill extracted mitochondrial genes. Table S2. KEGG2Gene of the *E. superba* extracted mitochondrial genes. Table S3. SSRs extracted from *E. superb* reported transcriptome sequences and part of our genome raw reads. Table S4. Collection of reported AMPs. Table S5. Putative AMPs identified from the *E. superba* and *P. vannamei* transcriptomes. Table S6. Summary of the identified AMPs/AMP precursors in both crustacean species with transcription levels (FPKM values). Table S7. Specific alignments of all mapped proteins and their corresponding matched AHTPs in both crustacean species. Table S8. Identified proteins with hit numbers of matched AHTPs in the Antarctic krill and whiteleg shrimp. Table S9. Number of mapped AHTPs in both crustacean species from transcriptome or genome data.
