# Supplementary material for: The First Genome Survey of the Antarctic Krill (Euphausia superba) Provides a Valuable Genetic Resource for Polar Biomedical Research
Source: Mar Drugs. 2020 Mar 31;18(4):185. doi: 10.3390/md18040185 (PMC7230668; doi:10.3390/md18040185)
Supplement: Supplementary file 1 [file marinedrugs-18-00185-s001.zip › Supplementary materials/Figure S1.docx]

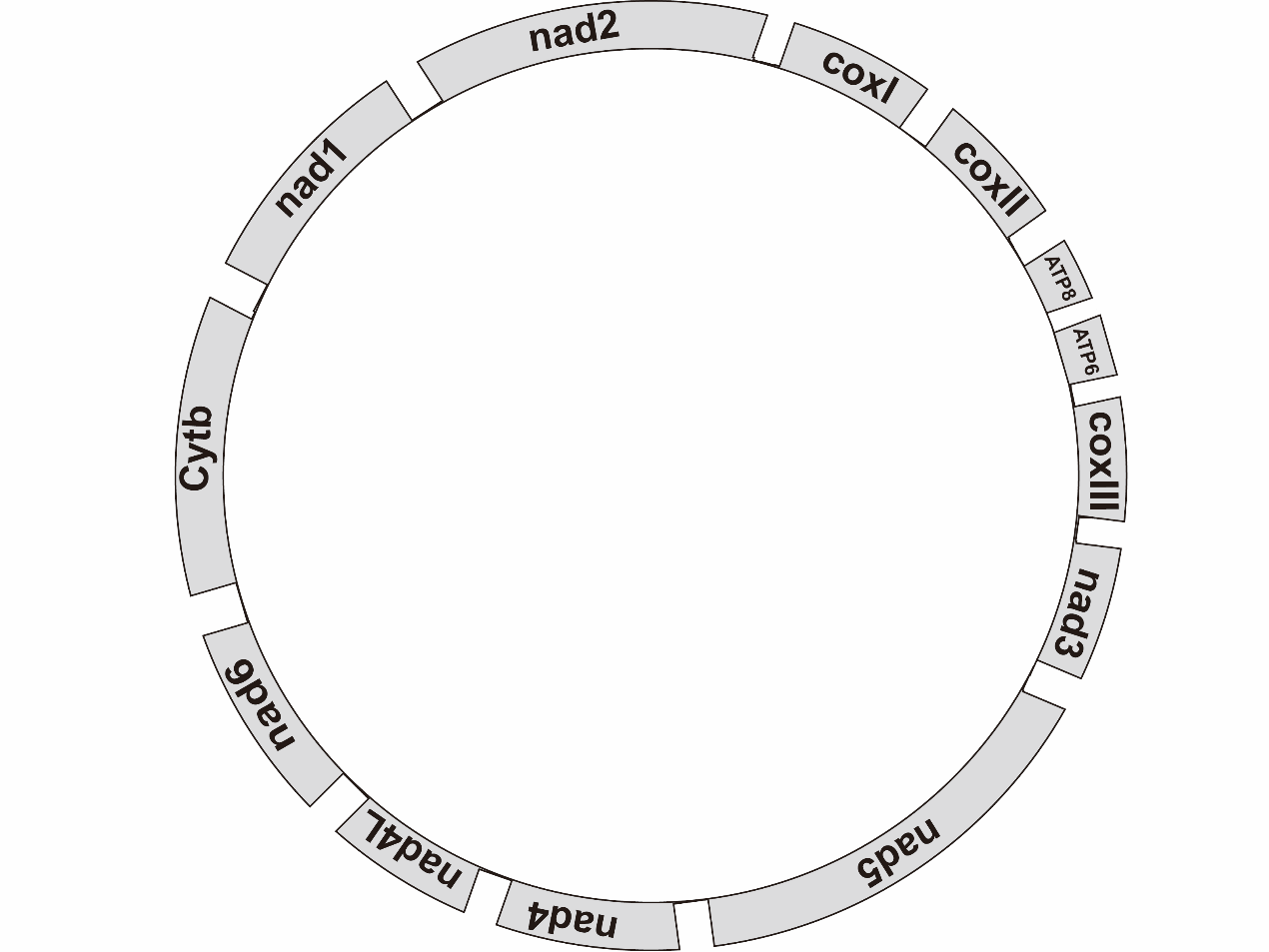


**Figure S1.** A sketch map for the 13 mitochondrial protein-coding genes of the Antarctic krill. The orders of these genes are in a clockwise direction, except for *nad1*, *nad4*, *nad4L*, and *nad5* [6].
