# Supplementary material for: The First Genome Survey of the Antarctic Krill (Euphausia superba) Provides a Valuable Genetic Resource for Polar Biomedical Research
Source: Mar Drugs. 2020 Mar 31;18(4):185. doi: 10.3390/md18040185 (PMC7230668; doi:10.3390/md18040185)
Supplement: Supplementary file 1 [file marinedrugs-18-00185-s001.zip › Supplementary materials/Figure S2.docx]

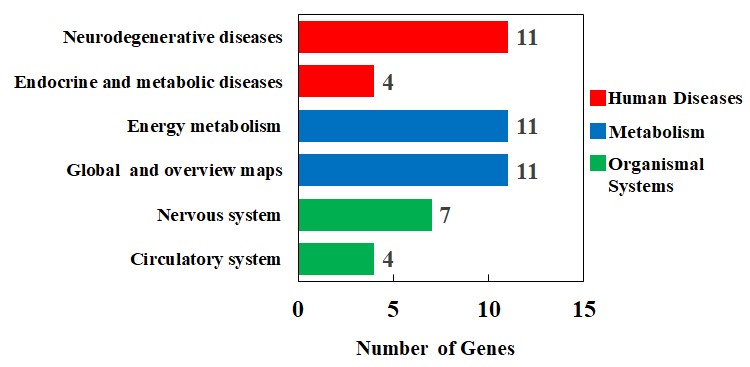

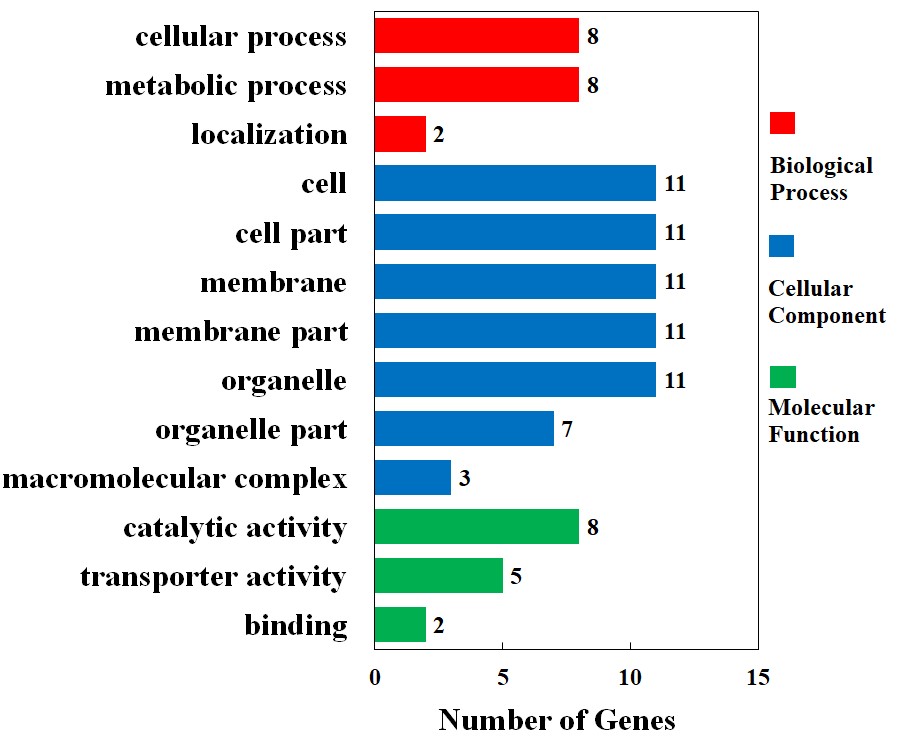


**Figure S2.** Functional classification of the *E. superba* mitochondrial genome. (**A**) KEGG pathway annotation of the mitochondrial genes. (**B**) Histogram of the GO terms.
