# Supplementary material for: The First Genome Survey of the Antarctic Krill (Euphausia superba) Provides a Valuable Genetic Resource for Polar Biomedical Research
Source: Mar Drugs. 2020 Mar 31;18(4):185. doi: 10.3390/md18040185 (PMC7230668; doi:10.3390/md18040185)
Supplement: Supplementary file 1 [file marinedrugs-18-00185-s001.zip › Supplementary materials/Figure S3 revised.docx]

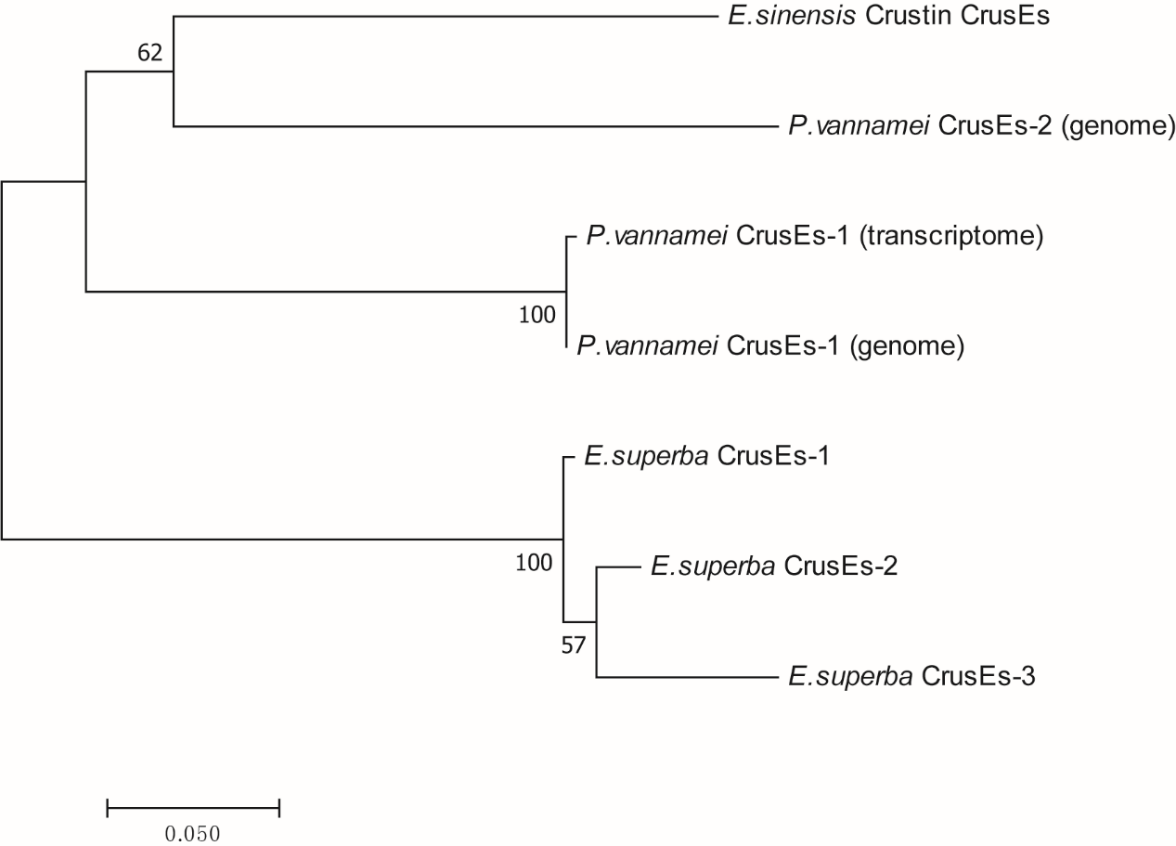


**Figure S3.** A phylogenetic analysis of the representative AMP precursors, CrusEs, from both crustaceans. The bootstrap test employed 1,000 replicates, and the numbers next to branches were replicate percentage of taxa clustering [22]. Corresponding amino acid sequences were analyzed in MEGA7 [23]. The CrusE sequence of Chinese mitten crab (*Eriocheir sinensis*; APD ID: AP01555) was used as an out-group. Please note that these data are corresponding to those in the Figure 7, using the same potein sequnces for detailed analysis.
