# Supplementary material for: The First Genome Survey of the Antarctic Krill (Euphausia superba) Provides a Valuable Genetic Resource for Polar Biomedical Research
Source: Mar Drugs. 2020 Mar 31;18(4):185. doi: 10.3390/md18040185 (PMC7230668; doi:10.3390/md18040185)
Supplement: Supplementary file 1 [file marinedrugs-18-00185-s001.zip › Supplementary materials/File S1.docx]

>nad2 [5144..6139]

ATTGTGTTTTCCCCTTCTCGTTGTTTGTTTATAAGAACGCTGATCTTAGGTATCACAATCTCAATTTCATCAAACTCCTGATTCGGAGCTTGAATAGGTTTAGAATTAAATCTACTCTCTTTTATCCCCTTAATTTTTATAAAAGATAACCAGTATTCGTCTGAAGCCGCCTTAAAATACTTCTTAATCCAAGCATTGGGATCAACCGTTTTATTGATTTCCGCTGTAATAATTATAATAAAGATACAGGTATTTTCTGCCCCATTGTCAGCTGCTTTATTGCTTAAAGCAGGCTCTGCGCCCTTTCATTTTTGATTTCCGCCAACCCTAGAAGGAATGATATGACCGCAAGCGTTAGTGTTAATGACAATCCAGAAAATTACACCAATATCACTATTATCTTATTACATCGAAGACCAAATAATACTGATCTCAGTCGCAGCCATAATATCGGCTGTAGTAGGAGCTCTAGGGGGGTTAAACCAAACCCTTCTACGAAAACTTCTTGGTTACTCATCAATTAACCACATAGCTTGAATAATATCGGCACTCCTGATAAGAGAAAGCATGTGGTTATTATACTTTGCTGTCTATTCTGTTATATCCTTTTCAGTAGTTATGCTCTTCCACTTTACACAGACCTTCCACTTTAACCATTTGTTAAATCACATTCCACCCGGTTCAGCAGTTAAAATAGTTATGTTTATAAGTCTTTTATCTCTAGGTGGTCTACCGCCTTTTACAGGATTTGTCCCAAAATGAATTGTTATTCAGCAATTATGCTGATCAAGTAACTTTTTAATGTTAACAATCCTTCTAGGTTCGTCTTTATTTACGCTCTTTTACTATCTACGAGTTACACTAAGCTCGCTTGTCCTAAGCTCCGCTAAGACTAAGTGGTCTATCAAGGTCGCTCTCAAGAGAAAACTTGTTATTATAGCCATGTTCGTCAATATAGTCGGACTACTAGTGCCATCAGCTGCTTATTCTTTTATA

>cox1 [6375..6872]

ACGCAGCAGCGTTGGCTATTCTCAACTAATCACAAAGACATTGGTACATTATACTTTATTTTCGGTGCATGAGCTGGGATAGTAGGTACTTCACTAAGATTGATTATTCGAGCTGAGTTAGGACAACCAGGTAGTTTAATTGGAGATGACCAAATTTATAATGTTGTAGTTACAGCACATGCTTTTGTTATAATCTTCTTTATGGTAATACCAATTATGATTGGTGGGTTTGGTAACTGGCTTGTTCCACTAATGCTAGGAGCCCCTGATATGGCATTCCCACGAATAAACAACATAAGATTTTGATTGTTACCCCCTTCCTTAACTCTCTTATTAGGAAGAGGTTTAGTAGAAAGTGGGGTTGGTACTGGGTGAACAGTATATCCACCTTTATCAGCAGGAATCGCTCATGCTGGAGCCTCTGTTGATATAGGAATCTTCTCGCTTCATATTGCCGGTGCTTCTTCAATTTTACTTAATTTTATACCAAGGATAGGC

>cox2 [11601..11825]

TATAAACTCTACTTCTAGAAAGTCTGAATATTCATAGCTTCAGTATCATTGATGTCCAATAGTTTTAAGAGTCACTCTGGGGTTATTAACTTCGTCTAATATATATAATAGTCGGAGAGATGGTAGAGCAATAAAAATAAGAATAATAGCCGGTACGACAGTTCAAATAATTTCGATTGTTTGACCTTCTAGTAGGAATCGATTTGTAATAGAATTAAAAAATAA

>atp8 [10944..11102]

TTATCATTTTCAAAATATTTTAGTGTAAGTTAACTTAGATTGAGTTGATGTAAGTTTGGAAGGAACTTTTATGAAGTAATTTACAGTTATAAATACTATAAATGTGGTTGAAAATATGATAAATAGATTTAGTCATAATAATGGTGATATTTGTGGGAT

>atp6 [10962..11177]

TTTAGTGTAAGTTAACTTAGATTGAGTTGATGTAAGTTTGGAAGGAACTTTTATGAAGTAATTTACAGTTATAAATACTATAAATGTGGTTGAAAATATGATAAATAGATTTAGTCATAATAATGGTGATATTTGTGGGATTAAAAAATGTTGCTTAAAGCGACAATTTAAGCTTGACAAGCTTATGTTAAAAACTGAAAATAAGTTTGTTATCAT

>cox3 [8489..8782]

TTAACTATTATTCTTGGGCTTTACTTTACTGCTCTGCAAGCATATGAGTATATTGAAGCGTCTTTCACAATTGCCGACTCTGTATACGGAGCTACTTTTTTTGTTGCCACAGGATTTCATGGCCTCCATGTAGTTATTGGAACTAGCTTCCTAGCTGTTTGTTTATTCCGTCTATATAGCTGTCACTTTTCAGCCAGCCACCACTTCGGATTTGAAGCAGCTGCATGATACTGACACTTTGTAGATGTTGTATGGTTATTTCTCTATATCTCTATCTATTGATGAGGAGGTTGT

>nad3 [8899..9171]

GTCATAACAATTGCATCTATTCTATCCAAAAAGACCATTTCAGACCGAGAAAAAAACTCCCCATTTGAATGCGGTTTTGACCCTAAAGGTTCAGCCCGCTTACCTTTCTCACTACGATTCTTCCTAATTGCAGTCATCTTTTTAATCTTTGATGTAGAGATTACACTACTTCTTCCTCTCGCTTCAATTATTGAAGTGTCTAATATCCAATCCTGAGTTACCACAGGAATTGTATTTTTGTTAGTATTATTGCTAGGTCTCTACCACGAGTGC

>nad5 [7245..8489]

ATGCTTTGACTTATCATATTTCACTTAAGAAGTATAGTTTTTTTAGTTTTGTTGTCGTGTAGTTGTATAATCAGGTCTATTTTTATAATTATAACTGATATAAGTTATTTTATTGAATGACATATTGTGAGAATTAATTCTTGTTCTATTGTTATAACTTTAATCTTAGATTGGATATCTCTCATATTTATAAGGTTTGTAAGATTTATTTCTTCTATAGTTTTATTTTACACTGGGGGTTATATAGACGGAGATATAAATATTAATCGTTTTATTTATCTCGTATTAGCTTTTGTAGCATCTATGGGATTTTTAATTATCAGTCCAAATATAGTAAGTATTCTTTTAGGTTGAGATGGGTTAGGGTTAGTGTCATATGTTTTAGTAATTTATTATCAAAATGAAAAATCTGCAAACGCTGGTATACTTACAGCACTTTCTAATCGTATTGGGGATGTCGCTATTTTGCTTGCTATTAGATTATTAGCTTCTCGTGGGGGCTGGAACTTTTATTTTTATAATGATTTATTCAATAATATAGAAGGTTCTGGTATTGTAGGTATATTAGTCGTGTTAGCTGCAATAACTAAGAGAGCTCAGATTCCATTTTCTGCATGATTACCTGCAGCAATAGCAGCTCCTACACCAGTGTCGGCTTTAGTCCACTCATCTACACTAGTAACTGCAGGAGTATACCTTCTAGTACGATTTAGGTCTGCAATTGAGGGCTCTATAACTCAGACTGTTCTTTTACTATTATCTAGGTTAACAATATTTATAGCAGGGTTGGGGGCTAATTTTGAATATGATTTAAAAAAAATTATTGCTTTATCTACTTTGAGACAGCTTGGTGTAATAATGAGAATTTTATCTTTAGGGTATGCTAACTTAGCTTTTTTCCACCTACTCAGGCACGCTTTATTTAAGGCACTGTTGTTTATATGTGCTGGGGCTGTAATTCATAATATAAAAGATTATCAAGATATTCGAGTTATGGGTAGTTTAGTTGTTCAAATACCTTTAACTACCTTTTGTATAAATTTGGCTAACTTAGCACTATGTGGAAGTCCATTCTTAGCTGGATTTTATTCTAAAGATCTAATTTTAGAAATTGCATTTATAAGGCCCATTAATATTATAATTTTTGTGTTATATTGCTTAGCAACAGGGTTAACTGTTTGTTATACAGCTCGTCTAATCTACTATACACTAAGAGGAGACTTTAATTTACATAGATTGTATACT

>nad4 [6846..7169]

TTACTTAATTTTATACCAAGGATAGGCCTCTGATGATTTTTACTAAGTGCTGGAAATATAGCAGCACCTCCAACTTTAAATTTATTAGGGGAGGTAAGTTTGATTATAAGGGTAGTGTCTTGATCTAAAGTAAGAATGATTATAATTGCATTTTTATCATTTTTTAGAGCTGCATATAGATTATATATGTATTCTCTAAGGCAACATGGTAAATATTACTCATCATTATTTTCTTGTTGTTCAGGGAAGGTGCGGGAATACTTGATTTTAATATTACATTGATTACCTTTAAATGCAATAATTCTTAATGGTTGTTTATTAGTA

>nad4L [4067..4366]

ATGTTAACACTTATATCTTATTACTTTGTTCCTATTTTAAGTGTATTATGCGGTCTGTGGGTTTTTTGCTCCAAGCGTAAACACTTATTGAATACTCTTCTGAGGCTAGAATATATTATACTGAGCGTATTTTGACTTATAGTACTAAGCTTATCATTAATAGGACAGGAAATCTTTTTCTCATTATTTTTTCTAACTTTTGCTGCTTGTGAGGGTGCTCTCGGGTTAGCATTACTAGTATCTATCGTTCGAAGTCATGGAAATGACCGATTTGGTAACTTTAATCTCTTACAATGTTAA

>nad6 [3406..3927]

TTAATTTGAAGATAGACGTAAAGGGCCAAAAAAGTTGCCTGTTACTTTAACCACAACAATAAGAGTTAAAAGCAAGTAAAAAACTATAAAGATAGTTAAATTTATATTAGTTGAATTATAAATAGTTGAAACTAACTCAAGATCTGTAGAGTAATAAAAATCCGTTATAAAAGAACTTATTTTTAGTAAAGTATTCTGTGATAAAATTATGGGGTCACAAATTAATGCTACGGCCCCAGTAACTGAAACTATTACAATTGCTATAAGCGATAGGATAGAAGGCTTGAACATTTCATTGGATGCTAAAGAGGTTACATAAATAAATAATACTAATATACCTCCTAAAAAAATGAGAAATAAAATATAAGAAAACCAGAATGATAAGTTAGATAAACCAGTGGTACAACAGATAAGGATAGTCTGGAACAATAATGCAAGTCCTATTGCAAGTGGGTGAACCAGTCGTGTAAATAAAATAGAGGTTGAAATAATTAAAGGATAAAATATAATTAAAAATGTAAT

>cob [2537..3406]

TTTAAGCTTATTTTCATTATATCAGCGGATTGCATAAGCAAATAAAAAGTATCATTCTGGTTGAATATGAGCTGGAGTAACCAAAGGGTTGGCAGGAATGAAGTTATCTGGGTCTCCAAGTAGATAAGGGTCAAGTAGAGTTAACAATAAAAGAGATGCTATTATAACTAAAATACCAAAAACATCTTTGAGAGTAAAATAATGGTGGAATGGGATTTTATCAATATTGTTTGGGAGACCAAGGGGGTTGTTAGAACCTGTTTGGTGTAAAAATAATAAATGAACTATCACAGCGGCGGCAACAATAAATGGAAATAAAAAATGGAAAGTAAAAAACCGAGTTAGAGTTGCATTATCTACAGCAAACCCTCCTCATAGTCACTGCACTAAGTCAGTTCCAATATAAGGAATAGCAGAAGCTAAATTTGTAATAACTGTTGCTCCCCAAAAAGATATTTGACCTCATGGTAAAACATATCCTAGGAAAGCTGTAGCCATTACTAAGAATATAATAATAACTCCAATAGTTCAGGTTTGTAAAAATAAATAAGAACCATAGTATATACCTCGTCCAGTGTGTAAATAAAGACAAATGAAGAAGAAAGAAGCTCCGTTGGCATGCATAGTACGAAGAAGTCAACCATAGTTAACATCTCGGCAAATGTGAGCAACACTAGAAAAAGCTAAATCAATATTGGCAGTATAATGTATTGCTAAAAATAAACCTGTAACAATCTGAACTCCTAAACAAAGTCCTAGTAGTGAACCAAAGTTTCACCACGCAGAAATATTAGACGGAGCAGGAAGGTCAACCAGTGCTCTATTTGCGATTTTAAATAAGGGATGATTTTTCCGGATTGGTATTGTCAT

>nad1 [2046..2567]

ATATCTTTTGTTATATTGATTAGTTATATTTTAGTTGTGATTTGTGTATTAGTTGGTGTAGCTTTTTTAACTTTATTAGAACGAAAAGTTCTTGGTTATATTCAAATCCGTAAAGGTCCTAATAAAGTTGGGTTTATTGGGATTTTACAACCTTTTTCTGATGCAATTAAACTATTTACAAAAGAACAAACATATCCTACGATATCAAATTTTATGCCGTATTATTTGTCTCCCGTGTTTAGGTTATTTGTATCATTAATTGTGTGATTAACTATGCCATACGAGATTGGTTTGTTTAATTTTTCAATAGGAACATTATTTTTTCTATGTTGTACAAGTTTAGGAGTTTATACTACAATGGGAGCGGGGTGATCATCAAATTCTAAGTATTCATTGTTAGGAAGTCTTCGAGCAGTAGCACAAACAATTTCATACGAAGTTAGATTAGCTTTGATTTTACTCTCTTTTATTATATTAGTTGGAGGGTTTAATTTAAGCTTATTTTCATTATATCAGCGGATT
