# Supplementary material for: The First Genome Survey of the Antarctic Krill (Euphausia superba) Provides a Valuable Genetic Resource for Polar Biomedical Research
Source: Mar Drugs. 2020 Mar 31;18(4):185. doi: 10.3390/md18040185 (PMC7230668; doi:10.3390/md18040185)
Supplement: Supplementary file 1 [file marinedrugs-18-00185-s001.zip › Supplementary materials/Table S9.docx]

**Table S9.** Number of mapped AHTPs in both crustacean species from transcriptome or genome data.

|  |  |  |  |
| --- | --- | --- | --- |
| **Antihypertensive**  **Peptides (AHTPs)** | ***Euphausi superba* (Transcriptome)** | ***Penaeus vannamei* (Transcriptome)** | ***Penaeus vannamei* (genome)** |
| AKYSY | 0 | 0 | 3 |
| GGY | 461 | 119 | 4,038 |
| GLP | 498 | 198 | 5,519 |
| IEKPP | 2 | 0 | 12 |
| IEP | 184 | 39 | 1,532 |
| IKW | 57 | 19 | 502 |
| IQW | 40 | 11 | 262 |
| IRPVQ | 0 | 0 | 11 |
| IRW | 28 | 5 | 327 |
| IRY | 140 | 30 | 957 |
| LGP | 371 | 99 | 3,883 |
| LIY | 196 | 39 | 1,533 |
| LKP | 442 | 92 | 3,817 |
| LRP | 311 | 70 | 4,658 |
| LRW | 61 | 16 | 1,025 |
| MAP | 198 | 37 | 1,625 |
| MDLA | 17 | 1 | 108 |
| MFDL | 8 | 1 | 78 |
| MKP | 103 | 21 | 904 |
| MLLCS | 0 | 0 | 5 |
| MLPAY | 0 | 0 | 5 |
| MRW | 18 | 5 | 237 |
| RYLGY | 0 | 0 | 6 |
| VFPS | 24 | 3 | 249 |
| VPAAPPK | 0 | 0 | 1 |
| VRF | 138 | 55 | 1,596 |
| VRKGQ | 1 | 0 | 7 |
| VSV | 519 | 139 | 6,220 |
| VVGAK | 1 | 0 | 13 |
|  |  |  |  |
